# Foreign Body Lodged in the Cheek for Ten Months

**Published:** 1848-10

**Authors:** 


*Foreign Body lodged in the Cheek for ten months.*—Professor South says, in his notes to the *translation of Chelius,* part xii, I have very recently operated on a case in which a piece of tobacco-pipe, an inch and a half long, had been lodged in the cheek for ten months, without the patient being aware of it. He had fallen with his pipe in hand, and wounded the outside of his cheek; much swelling ensued, and after a few weeks the wound healed, but he could not open his mouth completely nor without pain. Twice during the following twelvemonth, the swelling, which still remained, became very painful, increased and suppurated. The last time he came to me, and the piece of pipe was readily discovered running from half an inch behind the angle of the mouth horizontally back towards the angle of the jaw. I cut open the scar, which was very apparent, but had some little difficulty in detaching the end of the pipe, as the scar had probably sunk into its hollow; this done, however, it was easily drawn out with dressing forceps, like a dirk from its scabbard, and was evidently smeared with a mucoid secretion from the sides of the cavity which it had made for itself. In a few days the effects of the operation ceased: he opened his mouth freely and without pain, and the sinus filled up.

